# Time to full enteral feeds in hospitalised preterm and very low birth weight infants in Nigeria and Kenya

**DOI:** 10.1371/journal.pone.0277847

**Published:** 2024-03-08

**Authors:** Zainab O. Imam, Helen M. Nabwera, Olukemi O. Tongo, Pauline E. A. Andang’o, Isa Abdulkadir, Chinyere V. Ezeaka, Beatrice N. Ezenwa, Iretiola B. Fajolu, Martha K. Mwangome, Dominic D. Umoru, Abimbola E. Akindolire, Walter Otieno, Macrine Olwala, Grace M. Nalwa, Alison W. Talbert, Ismaela Abubakar, Nicholas D. Embleton, Stephen J. Allen, on behalf of the Neonatal Nutrition Network (NeoNuNet)

**Affiliations:** 1 Massey Street Children’s Hospital, Lagos Island, Lagos, Nigeria; 2 Department of Clinical Sciences, Liverpool School of Tropical Medicine, Liverpool, United Kingdom; 3 Alder Hey Children’s Hospital NHS Trust, Liverpool, United Kingdom; 4 Department of Paediatrics, University College Hospital, Ibadan, Nigeria; 5 Maseno University, Maseno, Kenya; 6 Department of Paediatrics, Ahmadu Bello University Teaching Hospital, Shika, Zaria, Nigeria; 7 College of Medicine, University of Lagos, Lagos, Nigeria; 8 Department of Paediatrics, Lagos University Teaching Hospital, Idi-Araba, Lagos, Nigeria; 9 Department of Clinical Research, KEMRI-Wellcome Trust Research Programme, Kilifi, Kenya; 10 Department of Paediatrics, Maitama District Hospital, Maitama, Abuja, Nigeria; 11 Department of Paediatrics, Jaramogi Oginga Odinga Teaching and Referral Hospital, Kisumu, Kenya; 12 Department of Paediatrics, Newcastle University, Newcastle upon Tyne, United Kingdom; 13 The Newcastle upon Tyne Hospitals NHS Foundation Trust, Newcastle upon Tyne, United Kingdom; Federal University of Sergipe, BRAZIL

## Abstract

**Background:**

Preterm (born < 37 weeks’ gestation) and very low birthweight (VLBW; <1.5kg) infants are at the greatest risk of morbidity and mortality within the first 28 days of life. Establishing full enteral feeds is a vital aspect of their clinical care. Evidence predominantly from high income countries shows that early and rapid advancement of feeds is safe and reduces length of hospital stay and adverse health outcomes. However, there are limited data on feeding practices and factors that influence the attainment of full enteral feeds among these vulnerable infants in sub-Saharan Africa.

**Aim:**

To identify factors that influence the time to full enteral feeds, defined as tolerance of 120ml/kg/day, in hospitalised preterm and VLBW infants in neonatal units in two sub-Saharan African countries.

**Methods:**

Demographic and clinical variables were collected for newborns admitted to 7 neonatal units in Nigeria and Kenya over 6-months. Multiple linear regression analysis was conducted to identify factors independently associated with time to full enteral feeds.

**Results:**

Of the 2280 newborn infants admitted, 484 were preterm and VLBW. Overall, 222/484 (45.8%) infants died with over half of the deaths (136/222; 61.7%) occurring before the first feed. The median (inter-quartile range) time to first feed was 46 (27, 72) hours of life and time to full enteral feeds (tFEF) was 8 (4.5,12) days with marked variation between neonatal units. Independent predictors of tFEF were time to first feed (unstandardised coefficient B 1.69; 95% CI 1.11 to 2.26; p value <0.001), gestational age (1.77; 0.72 to 2.81; <0.001), the occurrence of respiratory distress (-1.89; -3.50 to -0.79; <0.002) and necrotising enterocolitis (4.31; 1.00 to 7.62; <0.011).

**Conclusion:**

The use of standardised feeding guidelines may decrease variations in clinical practice, shorten tFEF and thereby improve preterm and VLBW outcomes.

## Introduction

Complications of prematurity (birth before 37 completed weeks) are the leading cause of neonatal and under-5 mortality [1], with the frequency and severity of morbidity and mortality increasing with decreasing gestational age (GA) and birthweight [2]. In a recent review of hospitalised newborns in Nigeria and Kenya, the leading risk factors for mortality were extreme prematurity (<28 weeks’ gestation) and very low birthweight (VLBW; <1.5kg) which increased the odds of mortality by 12 and 7 times respectively [3].

Early enteral feeding with breastmilk is vital in reducing adverse outcomes among hospitalised preterm VLBW infants. In addition to providing essential macro- and micronutrients, breast milk promotes the maturation of the gut microbiome which, in turn, promotes immune modulation, digestion and metabolism of feeds and neurodevelopment [4]. Early nutrition is therefore central to the prevention of short-term morbidities such as late-onset sepsis and feed intolerance with no evidence of an increase in necrotising enterocolitis (NEC); as well as promoting early infant growth, survival, and long-term optimal neurodevelopmental outcomes [5,6].

Enteral feeding and prematurity are the major risk factors for (NEC), a severe illness characterised by inflammation of the preterm infant gut leading to necrosis and/or perforation with a case fatality rate of 20–30% [7]. To prevent NEC, many clinicians delay the commencement of enteral feeds and/or advance feed volumes slowly for very preterm/VLBW infants, although there are few data to show this reduces the risk of NEC [8]. Despite the strict feeding protocols prescribed by Brown and Sweet [9], infection control measures and other practice changes made in neonatal intensive care, up to 7–10% of preterm infants in the United States and Canada continue to develop NEC which has encouraged further research into optimal feeding regimens [7,8,10].

In very preterm infants (<32 weeks’ gestation), the inability to coordinate suck, swallow and breathing and high risk of feeding intolerance and NEC make early feeding challenging [5]. In addition, breastmilk availability may be limited because of delayed lactogenesis due to maternal illness or a lack of adequate lactation support [11]. Early feeding for preterm infants may involve cup, spoon or naso- or orogastric tube feeding with graduated volumes of expressed mother’s own or donor breastmilk or artificial milk formula [5,12]. In some neonatal units (NNUs) in low-and-middle-income countries (LMICs), donor human milk may be obtained from a healthy wet-nurse [13], or from human-milk banks although this is more prevalent in high-income countries (HICs) [14]. Parenteral nutrition is often not available in LMICs, although intravenous fluids may still be used to provide energy and water until sufficient milk feeds are established [5].

The WHO infant feeding guideline group conducted systematic reviews comparing early versus delayed enteral feeding, and slow versus fast rates of feed advancement among preterm/VLBW infants, from which guidelines for optimal feeding of LBW infants in LMICs were developed [12]. They recommend that “VLBW infants in LMICs be given 10ml/kg/day of enteral feeds preferably starting from the first day of life, with the remaining fluid requirement met by intravenous fluids. Feed volumes can be increased by up to 30ml/kg/day with careful monitoring for feed intolerance.” A subsequent Cochrane systematic review [15] with bi-annual overviews of the systematic review [16–19] comparing the effect of slow (15-24ml/kg/day) versus fast (30-40ml/kg/day) enteral feed advancement have confirmed that rapid advancement of feeds does not increase the incidence of NEC or mortality among preterm VLBW infants. Despite this evidence-base, management of feeds among hospitalised preterm/VLBW infants varies within and outside sub-Saharan Africa, which may contribute to poor outcomes [11].

The time to full enteral feeds (tFEF) can be defined as the time it takes for the infant to tolerate an adequate volume of enteral feeds such that intravenous fluids or parenteral nutrition can be discontinued. It is a quantifiable and objective measure of feeding practice that captures the complex interactions between several factors including the baseline clinical status of the infant, the feeding guidelines being followed, and the morbidities the infant may develop during their admission. It is a sum of the effects of these factors on feed tolerance, encompassing what is prescribed versus what is tolerated by an infant. This study aimed to identify factors associated with the attainment of full enteral feeds in hospitalised preterm/VLBW infants across 7 neonatal units (NNUs) in Nigeria and Kenya.

## Methods

### Study design

Enteral feeding data of preterm, VLBW infants were collected prospectively in a multi-centre observational study (Neonatal Nutrition Network project, NeoNuNet; https://www.lstmed.ac.uk/nnu).

### Study setting

Details of the study setting have been described previously [3]. The 7 NNUs consist of two secondary and five tertiary level referral units, serving predominantly urban populations. They offer newborn care services to both inborn (born within participating perinatal centres) and outborn (born elsewhere then referred in) infants and provide newborn resuscitation, oxygen therapy, modified bubble continuous positive airway pressure (bCPAP), intravenous fluids, preterm enteral feeding, incubator care, intermittent and/or continuous Kangaroo Care, and phototherapy; none of the units routinely used CPAP machines or mechanical ventilators. The NNUs were selected based on existing research and clinical training collaborative partnerships between the Liverpool School of Tropical Medicine (LSTM) co-investigators and NNU leads in Nigeria and Kenya.

### Study population and sampling

Data on all newborn infants, aged <48hours, admitted into the NNUs over any 6-month period between September 2018 and April 2019 were collected, including data on patients with congenital anomalies and those who were products of multiple gestation. Data on newborn infants admitted at ≥48hours of life were excluded from this secondary analysis. Routine clinical information was collected until discharge, transfer, or death.

### Data collection and management

Standardised case record forms, co-developed by NeoNuNet co-investigators based on national and WHO neonatal clinical guidelines [3], were piloted at all sites and refined prior to data collection. Data recorded included maternal pregnancy/birth and neonatal characteristics, feeding practices and morbidity and mortality. Common morbidities (asphyxia, respiratory distress, sepsis, and abdominal conditions including feed intolerance and NEC) were diagnosed based on clinical features and results of laboratory and radiological investigations (where available).

Each NNU entered anonymised data into a Research Electronic Data Capture (REDCap) database, built and managed by a data manager at LSTM. All the data were exported into a.csv file to apply the INTERGROWTH standards (details below) to determine the appropriateness of growth for GA and sex and then imported into IBM Statistical Packages for Social Sciences software version 25 (SPSSv25) for analysis.

### Statistical analysis

For this study, moderate/late preterm was defined as GA between 32 and <37 weeks; very preterm as GA between 28 and <32 weeks, and extreme preterm as <28weeks. VLBW was defined as birthweight between 1.0 and 1.499kg and extreme low birthweight (ELBW) as birthweight < 1.0 kg; the term VLBW refers to both VLBW and ELBW unless otherwise stated. Small for gestational age (SGA) was defined as birthweight < 10th centile for GA and sex; appropriate for gestational age (AGA) as birthweight ≥ 10th centile including large-for-gestational age (LGA) infants with birthweight ≥ 90th centile. Stunting and wasting (asymmetrical SGA) were defined as length and weight/length ratio <3rd centile for GA and sex respectively. The anthropometric measures were compared to INTERGROWTH standards for weight, length and occipito-frontal circumference for boys and girls born at GAs between 24+^0^ and 42+^6^ weeks [20,21], and weight/length ratio between 33+^0^ to 42+^6^ [22]. For the weight/length ratio of babies born between 24+^0^ and 32+^6^ weeks, the z-scores and centiles were manually calculated using coefficients (means and standard deviations; see S1 Data) obtained from the INTERGROWTH-21st project team.

The tFEF in days was calculated as the difference between the day the infant tolerated 120ml/kg/day and the day of birth. The time to regain birthweight was the difference between the day the baby regained birthweight and the day of birth. Time to final outcome was computed as the difference between the date of discharge (equivalent to the length of hospital stay) or death and the date of birth.

Clinical variables were summarised as proportions for categorical variables and as medians and interquartile ranges (IQR) for continuous/interval variables. Pearson’s correlation was performed to test correlation between continuous interval measures while Mann-Whitney U and Kruskal-Wallis tests were used to test for univariate associations between tFEF and independent variables; the former for variables with 2 independent groups and the latter for variables with several independent groups. Multiple linear regression models were developed to control for confounding factors. A p value <0.05 was considered statistically significant and the effect sizes and 95% confidence intervals (95%CI) were calculated for independent predictors of tFEF.

### Ethics approval

Ethics approval for collection of anonymised patient data was granted by the LSTM Research and Ethics Committee and from the Institutional Ethics Committee for each NNU. The institutions and (Protocol Numbers) are as follows:

Liverpool School of Tropical Medicine (18–0210)

Jaramogi Oginga Odinga Teaching and Referral Hospital (ERC.IB/VOL.1/510)

University College Hospital, Ibadan (UI/EC/18/0446)

Massey Street Children’s Hospital (LSHSC/2222/VOL.VI^B^/185),

Ahmadu Bello University Teaching Hospital (ABUTH/HZ/HREC/D37/2018)

Maitama District Hospital (FHREC/2018/01/108/19-09-18)

Lagos University Teaching Hospital Health Research Ethics Committee (AMD/DCST/HREC/APP/2514)

Kenya Medical Research Institute-Scientific and Ethics Review Unit (KEMRI/SERU/CGMR-C/120/3740)

Individual consent was not obtained because data was analysed anonymously.

### Inclusivity in global research

Additional information regarding the ethical, cultural, and scientific considerations specific to inclusivity in global research is included in the Supporting Information (S1 Checklist).

## Results

Of the 2280 babies enrolled in the NeoNuNet database, 1172 (51.4%) were preterm and 484 (21.2%) were both preterm and VLBW.

### Neonatal characteristics

Of preterm/VLBW infants, 226 (46.7%) were male, 121 (25%) were moderate/late preterm and 249 (51.4%) were very preterm. The median (IQR) birthweight was 1.2 (1.1, 1.3) kg while the median GA was 30 (28, 32) weeks. A third of the preterm VLBW infants (117/393; 29.7%) were SGA, 42% of whom were disproportionately small (wasted) and about a fifth (73/393; 18.6%) were stunted; 53 and 85 infants were missing their birthweight and/or length at birth respectively. Classification by GA, birthweight, and appropriateness of growth for GA and sex are presented in S1 Table. Twenty-six (6%) LGA infants were included with the AGA group because of their small numbers. Fourteen (3%) babies had congenital abnormalities (S2 Table).

### Feeding practices

One hundred and thirty-six (28.0%) infants died before feeds could be commenced. Amongst the remaining 348 infants, median time to first feed was 46 (IQR: 27, 72) hours and only 29 (8.3%) infants were fed within the first hour of life. Mothers’ own milk was the most common type of first feed (270, 77.6%) then preterm formula (68, 19.5%); standard formula was rarely used (5, 1.4%). During admission, mother’s own milk remained the dominant type of feed; 203 infants were exclusively fed mothers’ own milk giving an exclusive breastfeeding rate of 58.3% but there was considerable mixed feeding (113; 32.5% infants). Twenty-four (6.9%) and 2 (0.6%) infants used preterm formula and standard formula exclusively respectively throughout the course of their admission.

Two hundred and forty-nine (71.6%) infants were initially tube fed via the orogastric/nasogastric route while 199 (57.2%) and 159 (45.7%) were fed by cup/cup-and-spoon and direct breastfeeding respectively.

**Fig 1:**
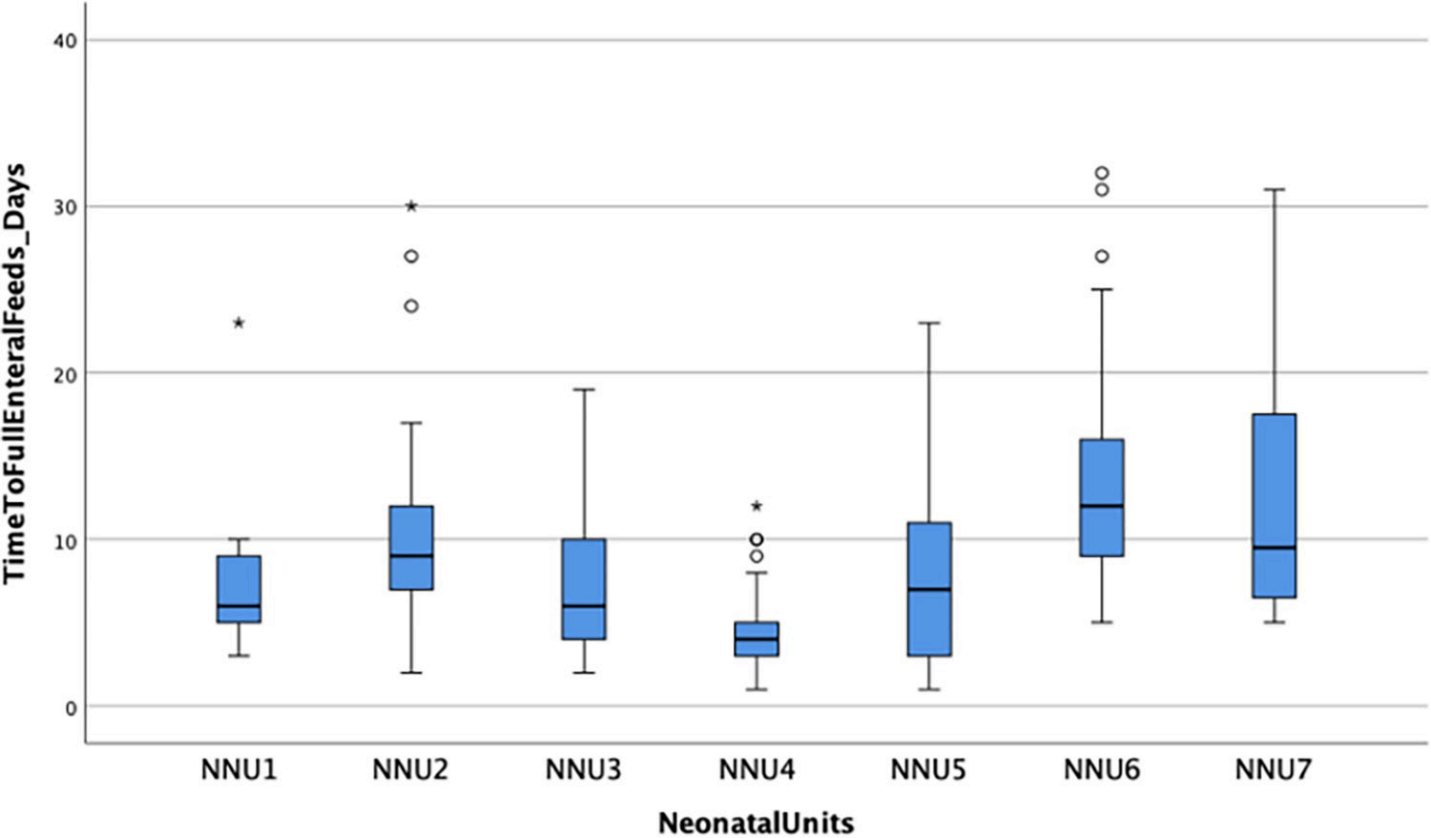
Boxplot of time to full enteral feeds by NNU. The horizontal line represents the median tFEF; the box represents the interquartile range; the whiskers represent the first and last quartiles of tFEF; represents outliers and * extreme outliers.

**Fig 2:**
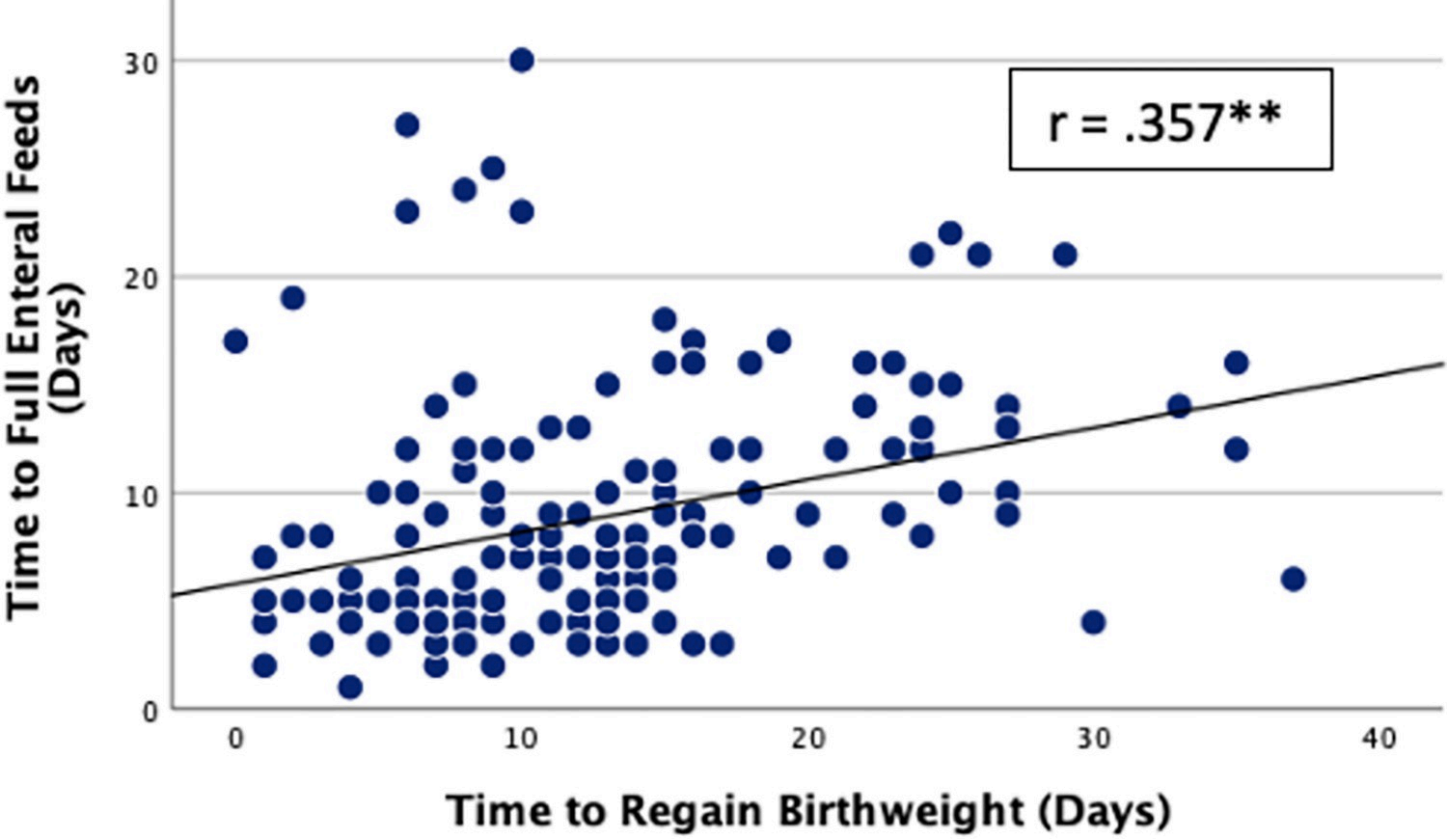
Correlation between tFEF and time to regain birthweight. ** Correlation significant at p value ≤0.01.

**Fig 3:**
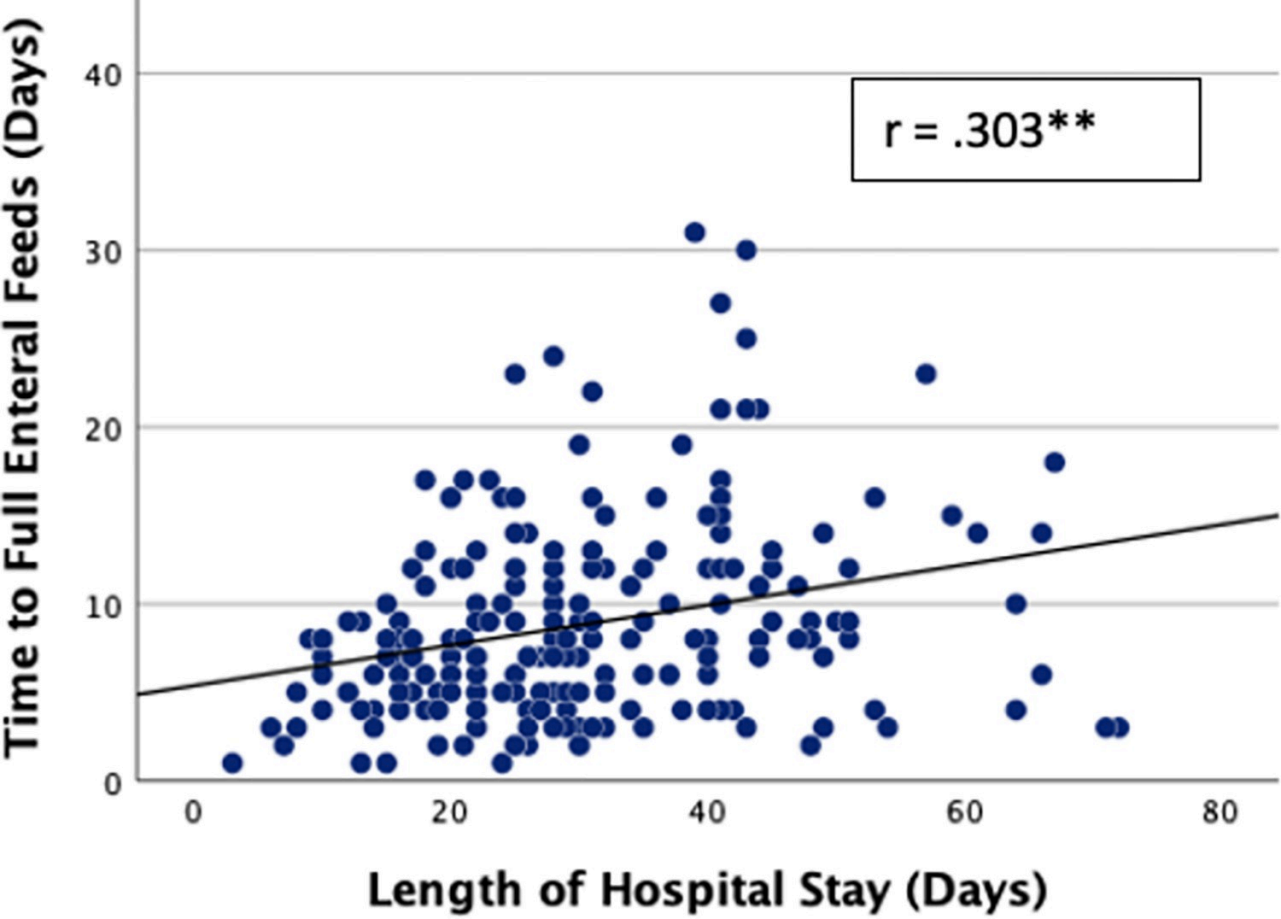
Correlation between tFEF and length of hospital stay. ** Correlation significant at p value ≤0.01.

**Fig 4:**
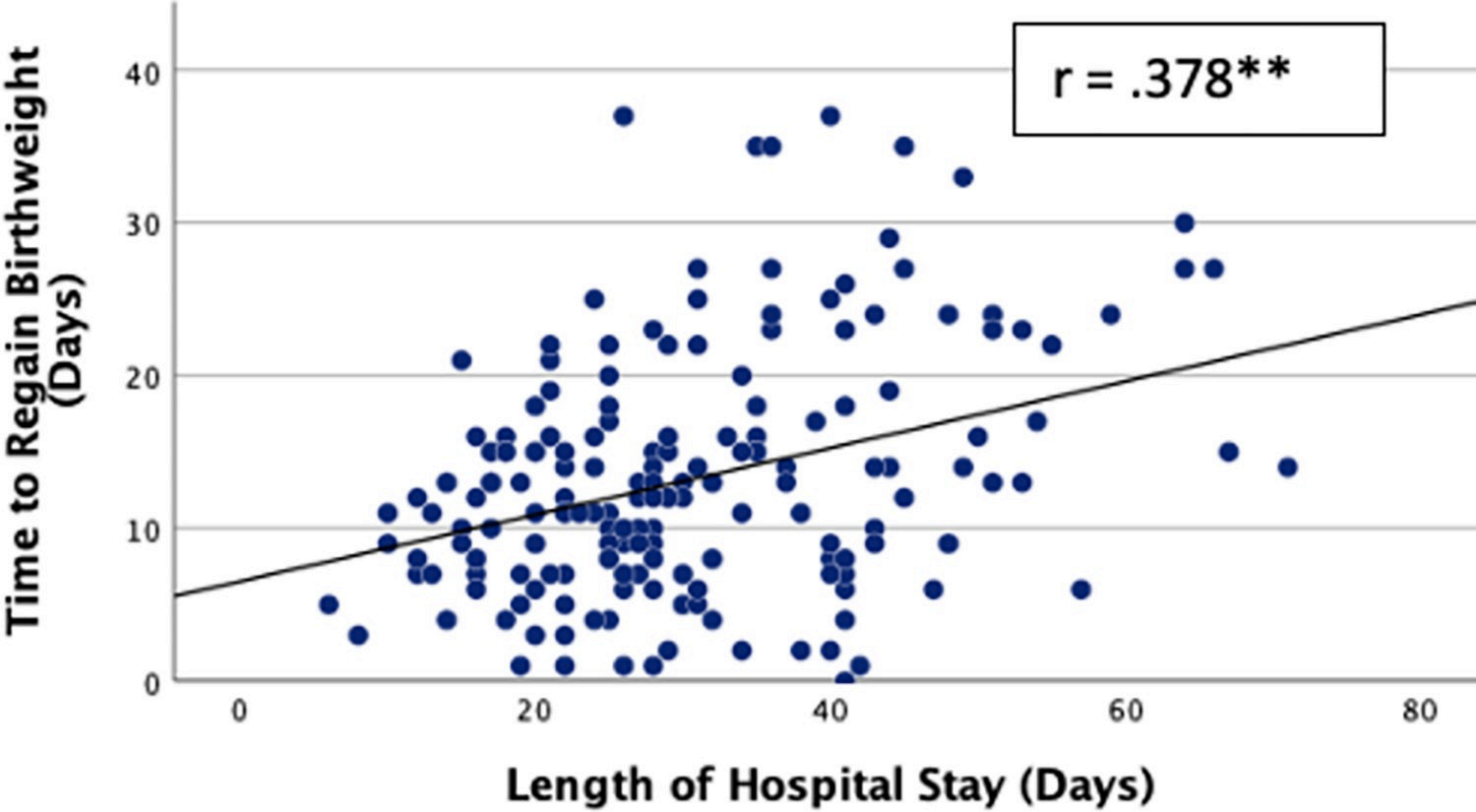
Correlation between time to regain birthweight and length of hospital stay. ** Correlation significant at p value ≤0.01.

Of the 348 infants who commenced feeds, 263 (75.6%) tolerated full enteral feeds (120ml/kg/day). Overall, full enteral feeds were established by a median time of 8 (4.5, 12) days but tFEF varied markedly between NNUs ranging between 1–32 days (Fig 1). Infants regained their birthweight by a median of 12 (7, 16) days while the median length of hospital stay among survivors was 27 (20, 38.5) days. There was a moderate positive correlation between tFEF and time to regain birthweight (r = 0.357, p < 0.001; Fig 2), and length of hospital stay (r = 0.303, p < 0.001; Fig 3). Time to regain birthweight also has a moderate, though greater, correlation with length of hospital stay (r = 0.378, p < 0.001; Fig 4).

Overall, only 92 (19%) infants from 4 NNUs in Nigeria received parenteral amino acid infusions; one NNU used them routinely with 74% of their preterm and VLBW infants receiving parenteral amino acids for an average of 7 days. Parenteral lipids were only used on one patient in Nigeria.

### Morbidity and mortality

Forty (8%) infants had perinatal asphyxia, 255 (53%) had varying degrees of respiratory distress, 217 (45%) developed sepsis and 22 (5%) developed abdominal conditions including feed intolerance and NEC. All the patients who had respiratory distress received oxygen therapy which lasted for a median (IQR) of 3 (2, 7.75) days. The overall mortality rate was 46% (222/484) with half of the deaths occurring by 2 days and three-quarters by 6 days (S1 Fig). Case fatality rates were 62.5%, 49.0%, 46.5% and 59.0% for asphyxia, respiratory distress, sepsis, and abdominal conditions respectively.

### Association between clinical variables and time to full enteral feeds

**Fig 5:**
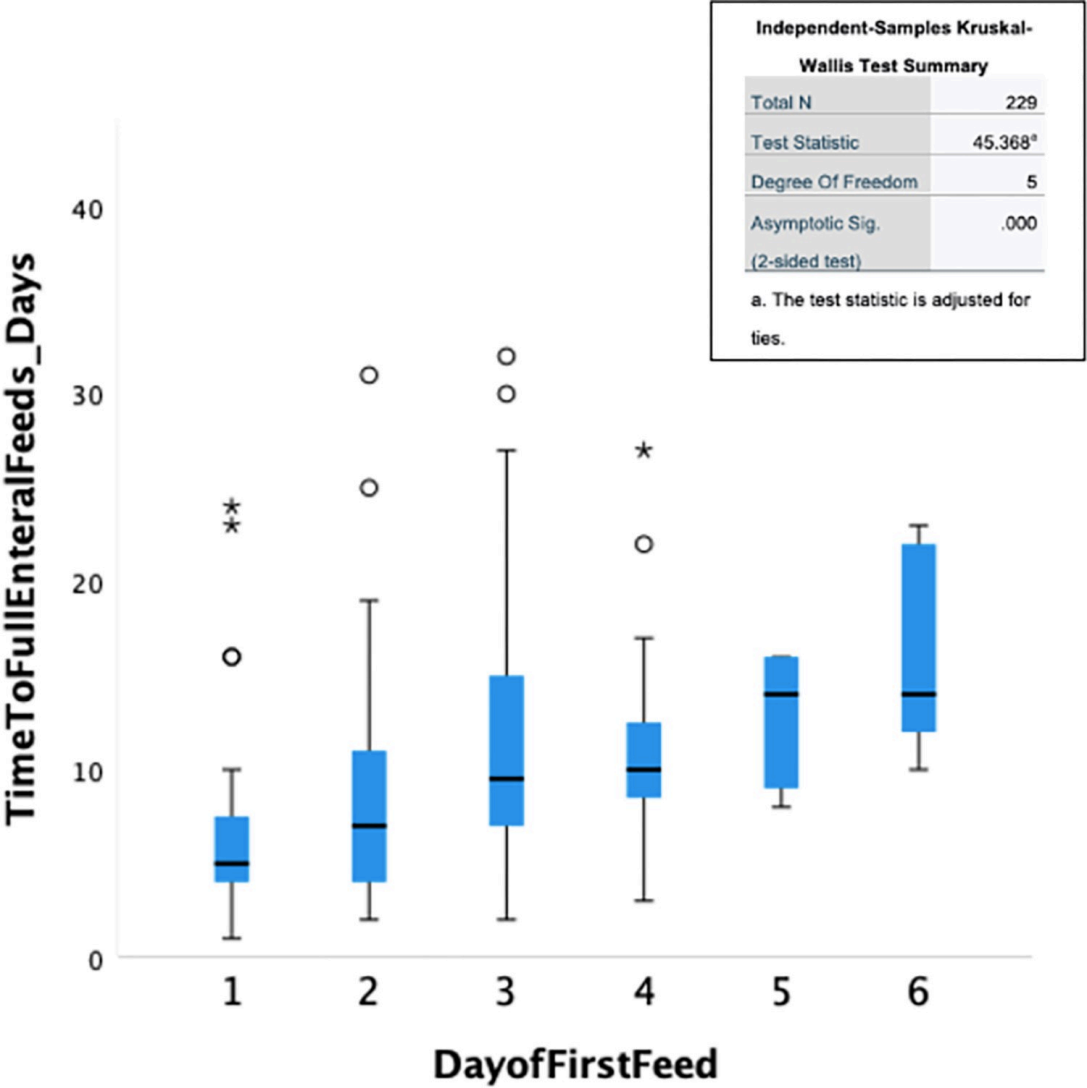
Median time to full enteral feeds according to day of first feed. The horizontal line represents the median tFEF; the box represents the interquartile range; the whiskers represent the first and last quartiles of tFEF; ° represents outliers and * extreme outliers.

In univariate analysis, starting feeds earlier was associated with a shorter time to establish FEF (P<0.001). In pairwise comparisons with adjusted p-values, there was a significant reduction in tFEF when feeds were commenced on the 1st day of life compared to the 3rd (p = 0.000, r = -0.57), 4th (p = 0.000, r = -0.48), 5^th^ (p = 0.013, r = -0.38) or 6th (p = 0.004, r = -0.42) days. There was no significant difference in tFEF between starting feeds on the 1st compared to the 2nd day of life (p = 0.660, r = -0.23; Fig 5). Data were not collected on the volume of initial feed or the rate of advancement. Gestational age was significantly associated with tFEF (p = 0.003) with a significant reduction in tFEF among moderate/late compared to very preterm infants on pairwise comparison with adjusted p values (p value = 0.002, r = -0.23); there was no significant difference in tFEF between very and extreme preterm infants (p = 1.00, r = 0.05) and the decrease in tFEF among moderate/late compared to extreme preterm infants did not reach significance (p = 0.235, r = -0.16). There was no association between type of feed, sex, or anthropometric indices and tFEF. Amongst common morbidities, the occurrence of an abdominal condition was associated with a longer tFEF (p = 0.004). Conversely, respiratory distress was associated with a statistically significant shorter tFEF (p = 0.001) (Table 1).

**Table 1:** Associations between demographic and clinical variables and time to full enteral feeds; univariate analysis.

Variable	Median tFEF (IQR) in Days	p value	Effect size (r)
**Sex**			
**• Female**	7.5 (4.3, 12)	0.976	-0.002
**• Male**	8 (5, 12)		
**By GA**			
**• 32-<37 weeks**	6 (4, 9)		
**• 28 <32weeks**	8 (6, 12)	**0**.003*	0.16
**• <28 weeks**	8 (4,12)		
**By Birthweight**			
**• 1–1.5kg**	8 (5, 12)	0.482	0.05
**• <1kg**	8 (5, 14)		
**Weight Centiles**			
**• AGA**	8 (5, 12)	0.933	0.006
**• SGA**	8 (5, 12)		
**Length Centiles**			
**• Not Stunted**	8 (3, 13)	0.107	-0.114
**• Stunted**	7 (4, 11)		
**Weight/Length Ratio Centiles**			
**• Not Wasted**	8 (6, 13)	0.200	-0.090
**• Wasted**	7 (4,11)		
**First Feed: colostrum/breastmilk**			
**• Yes**	7 (4, 12)	0.148	0.091
**• No**	9 (6, 12)		
**First Feed: Preterm Formula**			
**• No**	7 (4, 12)	0.312	0.064
**• Yes**	9 (5, 12)		
**First Feed: Standard Formula**			
**• No**	8 (4, 12)	0.374	0.056
**• Yes**	5.5 (5.3, 8)		
**Asphyxia**			
**• No**	8 (5,12)	0.610	-0.032
**• Yes**	7 (4, 10)		
**Respiratory Distress**			
**• No**	8 (5, 13)	**0.001** *	0.201
**• Yes**	7 (4, 10)		
**Sepsis**			
**• No**	8 (4, 12)	0.822	-0.014
**• Yes**	7 (5, 10)		
**Abdominal Conditions**			
**• No**	7 (4, 11)	**0.004** *	0.182
**• Yes**	16 (7.5, 24.5)		

tFEF = time to full enteral feeds; IQR interquartile range; GA = gestational age; AGA = appropriate for gestational age; SGA = small for gestational age

* significant p value.

Variables that were found to be independent predictors of tFEF on multiple linear regression analysis were day of first feed (unstandardised B 1.69; 1.11, 2.26; p <0.001), GA (unstandardised B 1.77; 95%CI 0.72, 2.81; p <0.001), respiratory distress -2.14 (-3.50, -0.79; p = 0.002) and abdominal conditions including NEC 4.314 (1.00, 7.62; p = 0.011 (Table 2). This translates to tFEF increasing by (i) 2 days for every 24-hour delay in commencing feeds (after the first 24hours); (ii) 1–2 days for very- and extremely preterm infants compared to moderate/late preterm infants; and (iii) 5 days for every day on nil per oral for abdominal conditions including NEC. This also translates to tFEF decreasing by at least 2 days for everyday on oxygen therapy for respiratory distress. Each interpretation is only true if all other predictors are held constant.

**Table 2:** Associations between demographic and clinical variables and time to full enteral feeds; multiple linear regression analysis.

Predictor Variable	Unstandardised Coefficient B (95% CI)	p Value
**Constant**	2.71 (0.27, 5.15)	0.030
**Day of first feed**	1.69 (1.11, 2.26)	<0.001
**Gestational age**	1.77 (0.72, 2.81)	<0.001
**Respiratory distress**	-2.14 (-3.50, -0.79)	0.002
**Abdominal conditions**	4.31 (1.00, 7.62)	0.011

R^2^ = 0.227; p value <0.001.

## Discussion

In this analysis, tFEF was significantly associated with time of first feed, the gestational age of the preterm infant and the occurrence of respiratory distress and abdominal conditions on multiple regression.

The NeoNuNet defined full enteral feeds as 120ml/kg/day, where other studies have defined full enteral feeds as 120 and 150 ml/kg/day [11], 140 ml/kg/day [23] or 150-160ml/kg/day [24]. The overall median tFEF in this study was 8 (IQR: 4.5,12) days not dissimilar to that reported by de Waard et al which ranged from 8–33 days for full enteral feeds defined as 120ml/kg/day [11]; their analysis included data from 13 NNUs, only one of which was from Nigeria, and the median tFEF for that sole Nigerian hospital was 11 days.

This analysis confirms the finding of other studies that the later feed is commenced, the later full enteral feeds (FEF) is attained [11]. Feeds were commenced on the second or third day of life across the 7 participating NNUs. Enteral feeds may be delayed because mothers’ own milk is not available, due to the clinical status of the newborn or as a unit practice. This analysis did not find any difference in tFEF among infants who were commenced on mothers’ own milk, preterm formula, or standard formula. Kreissl et al, working in Vienna, comparing prospective observational data with retrospective medical records, found a decrease of 4 days in tFEF when feeds were commenced with single donor human milk compared to preterm formula; both groups commenced feeds on the first day life [23]. In the de Waard study, median tFEF was 26 days (for 120ml/kg/day) in 5 NNUs in South China that used infant formula predominantly, feeds were commenced between the 2^nd^ to 5^th^ day of life and feed advancement was conservative [11]. As reported by other studies that the severity and frequency of complications of prematurity (including feed intolerance and susceptibility to NEC) increase with decreasing GA [2,3,5], this study finds that the attainment of full enteral feeds was delayed with increasing prematurity or decreasing GA. The very and extreme preterm infants are also more likely to have the initiation of feeds delayed for fear of them developing NEC [8,11,13]. There was no difference in tFEF based on birthweight alone and, although 30% of the preterm VLBW infants were SGA, this was not associated with tFEF; infants who were wasted (asymmetric SGA) and those who were stunted tended to have shorter tFEF the difference did not reach significance.

Clinical conditions that could delay the commencement of feeds include the need for bag-and-mask ventilation, perinatal asphyxia, or respiratory distress. Perinatal asphyxia did not have a significant effect on tFEF in this analysis but interestingly, infants who had respiratory distress attained FEF significantly earlier than those who did not. Although, theoretically, this may be because the infants with respiratory distress were placed on oxygen therapy and the improved oxygenation (including intestinal oxygenation) improved feed tolerance and hastened the attainment of full enteral feeds, this finding is more likely due to non-differential misclassification bias from respiratory distress being defined by its occurrence. If respiratory distress had been defined as distress persisting/persistent beyond the first 48 hours of life (excluding transient tachypnoea of the newborn which would have resulted in the resolution of symptoms in time to commence feeds on the 2^nd^ or 3^rd^ day of life), the results may have been different.

In this study, infants who had feed intolerance and/or NEC attained full enteral feeds as much as 5 days later than those who did not. The clinical signs and management of feed intolerance and NEC necessitate the interruption and/or discontinuation of feeds in addition to the use of antibiotic therapy and surgical intervention [7] which would delay the achievement of full enteral feeds as was found in this analysis. This association of abdominal conditions with delay in attaining tFEF was significant despite the low incidence of this complication in this cohort. The low NEC rates may be because a third of the cohort died before they could be commenced on enteral feeds which is a key risk factor in the pathogenesis of NEC. It may also be because of the predominant use of mothers’ own milk in this study [7,8,10]. The de Waard study did not find a significant difference in NEC rates across NICUs in South China and Europe/Oceania/North America/Africa despite differences in feeding protocols, but it did not report on any statistical tests of association between tFEF and NEC or other short-term outcomes [11].

From the foregoing, feeds were most likely delayed as a result of the feeding practice in use in the participating NNUs. This finding is corroborated by a related survey conducted among neonatologists/paediatricians working in Nigeria and Kenya [13] which documented that feeds were initiated with and advanced at 10-20ml/kg/day and were given in 2–3 hourly boluses by over 70%, 80% and 100% of respondents respectively; it also documented that feeds were commenced after the first 24 hours of life by over 50% of respondents [13]—a practice informed by the fear of the preterm VLBW developing NEC [7,8,10], despite available evidence to the contrary. Evidence suggests these practices may result in up to 2 more cases of NEC, 3 more deaths and 15 more cases of feed intolerance, and it may also result in 5 to 6 more cases of sepsis per 100 VLBW infants [18,19]. Slow advancement also results in increased hospital stay of up to 6 days [18,19] which increases the direct and indirect cost of care particularly in LMICs where hospital fees are borne out-of-pocket [25]. Sub-group analyses based on the type of feed (breastmilk vs. formula vs. mixed feeds) or if the infants were ELBW (extreme low birth weight; <1kg) and/or SGA did not show a significant association with the incidence of NEC.

In summary, this study documents that the attainment of full enteral feeds among hospitalised preterm and VLBW infants is delayed across NNUs in Nigeria and Kenya and that the delay is most likely due to feeding practice in use in the NNUs and the development of abdominal conditions including feed intolerance and NEC. The unanticipated association of tFEF with respiratory distress requires further investigation. Further studies are required to evaluate the implementation of evidence-based feeding guidelines in NNUs across sub-Saharan Africa.

### Strengths

One of the strengths of this study is that it involved prospective data collection which allowed for a more objective and complete dataset. Another strength is the large number of preterm and VLBW infants recruited from NNUs providing different levels of care, in resource-limited settings in sub-Saharan Africa which may make the results more generalisable in this setting. The routine clinical data collected provides an opportunity to identify gaps in clinical care.

### Limitations

There were a few limitations with this analysis; the details on initial feed volume, the rate of advancement, whether tube feeds were given in boluses or continuously and the frequency of bolus feeds were not collected, and these may have also impacted on tFEF.

Another limitation was the variation in the numbers of patients recruited from each NNU which made it difficult to evaluate the true effects of birth anthropometry, GA, and morbidity on tFEF. A further study with standardised feeding protocols across these NNUs would provide greater certainty.

## Conclusion

Our findings among hospitalised preterm and VLBW infants admitted to NNUs in African settings strongly suggest that delays in initiating enteral feeds and the occurrence of abdominal conditions (including feed intolerance and NEC) are associated with delays in achieving full enteral feeds resulting in longer hospital stay. The effect of respiratory distress on time to full enteral feeds requires further investigation. The implementation of evidence-based, standardised feeding protocols is needed urgently with further research to evaluate their impact.

## Supporting information

S1 Checklist Inclusivity in global research questionnaire. (DOCX)

S1 Fig Time of death of infants. (TIF)

S1 Table Characteristics of Preterm and VLBW Infants. (PDF)

S2 Table Congenital Abnormalities. (PDF)

S1 Data NeoNuNet Time to full enteral feeds data. (XLSX)
