# Supplementary figures and images for: Time to full enteral feeds in hospitalised preterm and very low birth weight infants in Nigeria and Kenya
Source: PLoS One. 2024 Mar 8;19(3):e0277847. doi: 10.1371/journal.pone.0277847 (PMC10923414; doi:10.1371/journal.pone.0277847)

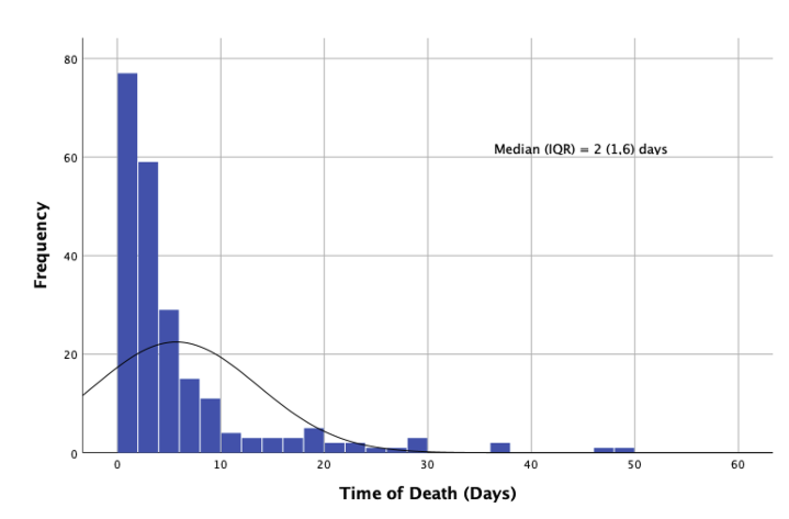

Supplement: S1 Fig — (TIF) [file pone.0277847.s002.tif]
