# Supplementary material for: Time to full enteral feeds in hospitalised preterm and very low birth weight infants in Nigeria and Kenya
Source: PLoS One. 2024 Mar 8;19(3):e0277847. doi: 10.1371/journal.pone.0277847 (PMC10923414; doi:10.1371/journal.pone.0277847)
Supplement: S1 Table — (PDF) [file pone.0277847.s003.pdf]

S1 Table: Characteristics of Preterm and VLBW Infants

	NNU1 13 (2.6)	NNU2 104 (21.5)	NNU3 58 (12.0)	NNU4 92 (19)	NNU5 66 (13.6)	NNU6 128 (26.4)	NNU7 23 (4.8)	Total 484 (100)
Male	8 (61.5)	52 (50.0)	26 (44.8)	45 (48.9)	32 (48.5)	52 (40.6)	11 (47.8)	226 (46.7)
Birthweight (kg)**	1.4 (1.2,1.4)	1.2 (1.0,1.3)	1.2 (0.9,1.3)	1.2 (1.0,1.4)	1.1 (1.0,1.2)	1.1 (1.0,1.3)	1.2 (1.0,1.4)	1.2 (1.1,1.3)
GA**	31 (30,32)	29 (28,31)	30 (28,32)	30 (27,32)	29 (28,30)	29 (27,31)	32 (31,33)	30 (28,32)
Birthweight††:								
• 1000 – 1499g	11 (84.6)	77 (74)	41 (70.7)	71 (77.2)	50 (75.8)	94 (73.4)	19 (82.6)	363 (78)
• < 1000g	0 (0)	17 (16.3)	15 (25.9)	20 (21.7)	16 (24.2)	34 (26.6)	3 (13.0)	105 (22)
GA:								
• 32 - <37wks	5 (38.5)	19 (18.3)	19 (32.8)	27 (29.3)	13 (19.7)	24 (18.8)	14 (60.9)	121 (25.0)
• 28 - <32wks	8 (61.5)	63 (60.6)	26 (44.8)	39 (42.4)	40 (60.6)	64 (50.0)	9 (39.1)	249 (51.4)
• < 28wks	0 (0)	22 (21.2)	13 (22.4)	26 (28.3)	13 (19.7)	40 (31.3)	0 (0)	114 (23.6)
Birthweight†:								
• <10th Centile	1 (9.1)	18 (19.4)	11 (40.7)	25 (29.4)	22 (33.8)	34 (26.6)	14 (63.6)	125 (29.0)
Birth Length††:								
• <3rd Centile	0 (0)	20 (19.4)	7 (25.9)	18 (35.3)	10 (16.4)	9 (7.0)	12 (52.2)	76 (19.0)
Weight/Length Ratio:								
• <3rd Centile	0 (0)	16 (15.5)	4 (14.8)	13 (25.5)	8 (13.1)	12 (9.4)	8 (34.8)	61 (15.2)
OFC:								
• <3rd Centile	0 (0)	7 (6.9)	6 (22.2)	10 (18.9)	6 (9.8)	2 (1.6)	7 (31.8)	38 (9.4)

VLBW = very low birth weight; GA = gestational age; OFC=occipito-frontal circumference

Data are number (%) or **median (IQR); † 53 missing birthweights; †† 85 missing birth lengths; 81 missing OFC.
