# Supplementary material for: Time to full enteral feeds in hospitalised preterm and very low birth weight infants in Nigeria and Kenya
Source: PLoS One. 2024 Mar 8;19(3):e0277847. doi: 10.1371/journal.pone.0277847 (PMC10923414; doi:10.1371/journal.pone.0277847)
Supplement: S2 Table — (PDF) [file pone.0277847.s004.pdf]

S2 Table: Congenital Abnormalities

Congenital Abnormalities	Frequency
Acyanotic Congenital Heart Disease	3
Hydrocephalus	2
Down Syndrome	2
Microcephaly	1
Duodenal atresia + Atrioventricular Cushion Defect + Patent Ductus Arteriosus	1
Oesophageal Atresia + Tracheo-oesophageal fistula	1
Gastroschisis	1
Jejunal Atresia	1
Congenital amputation of the right foot	1
Genu recurvatum	1
**Total**	**14 (2.8%)**
